# The Disease Spectrum and Influencing Factors of NICU in Xiangxi, Underdeveloped Area of China: A 9-Year Retrospective Study

**DOI:** 10.3389/fped.2022.874586

**Published:** 2022-06-17

**Authors:** Fen Xie, Yuhua Zhu, Lulu Chen, Ruyi Han, Qingxia Shu, Zheng-ying Chen, Jinxiu Li

**Affiliations:** ^1^Department of Nursing, Jishou University School of Medicine, Jishou, China; ^2^Department of Neonatology, The Fourth Affiliated Hospital of Jishou University (The First People's Hospital of Huaihua), Huaihua, China

**Keywords:** newborns, neonatal intensive care unit (NICU), disease spectrum, influencing factor, underdeveloped region

## Abstract

**Objective:**

Investigation of the basic conditions and disease spectrum in neonatal intensive care unit (NICU) from 2012 to 2020, in the underdeveloped area of Xiangxi, China.

**Methods:**

All newborns (*N* = 16,094) admitted to the NICU of a hospital in the Xiangxi area from 2012 to 2020 were selected for the retrospective study.

**Results:**

The average male/female ratio was 1.43:1, with 9,482 males and 6,612 females admitted to the NICU. The sample comprised 41.02% premature infants, and 56.52% had been delivered *via* cesarean delivery (CD). The most prevalent diseases diagnosed in the NICU were jaundice (22.01%), respiratory (18.45%) and neurological diseases (17.54%). Over the 9-year study window, the prevalence of jaundice and cardiovascular diseases increased, while respiratory and neurological diseases became less frequent. The prevalence of the remaining diseases remained unchanged. Prevalence of neonatal diseases is influenced by gender, patient sources, delivery methods, gestational age and birth weight (*P* < 0.05). The prevalence of neonatal diseases was significantly higher in males, infants born *via* CD, and in infants of lower gestational age and birth weight.

**Conclusion:**

The study contributes in-depth information about infant characteristics in an NICU in an undeveloped region of China. In the past 9 years, the average proportion of premature infants in the NICU decreased to 37.38% in 2020, but this figure remains higher than the Chinese national average of 26.2%. Similarly, the CD rate is higher than the Chinese average. The spectrum of neonatal diseases in the NICU in Xiangxi area is drawn, included jaundice, respiratory and neurological diseases, primarily. Through statistical analysis, it is found that the types and prevalence of neonatal diseases are closely related to different gender, gestational age, patient sources, delivery methods, and birth weight (*P* < 0.05). Newborns of specific gestational age, birth weight and delivery method should be considered “at-risk” and targeted in the formulation of preventive measures. There is a great need to improve the diagnosis and treatment of neonatal diseases—and perinatal health care in general—to ensure improved outcomes for newborns admitted to NICUs in underdeveloped regions.

## Introduction

The World Health Organization (WHO) reports that neonatal diseases constitute the fifth most common cause of death in the world (1), causing ~2.44 million newborn deaths every year (2). Investigations of neonatal diseases in underdeveloped areas are helpful to reduce the prevalence and mortality rates of neonatal diseases in such areas. Xiangxi is an underdeveloped area in China and a key region supported by the “Healthy China 2030 Plan.” Retrospective study of neonatal intensive care unit (NICU) diseases and its influencing factors in Xiangxi is beneficial for understanding NICU diseases in similar areas in China and can provide a theoretical basis for the prevention and treatment of neonatal diseases in NICUs in similar areas.

The “Healthy China 2030 Plan” reports that China faces new challenges and a changing disease spectrum (3). In 1990, the major diseases affecting Chinese newborns were neonatal pneumonia, sepsis and scleroderma, while 20 years later, in 2009, jaundice, pneumonia and hypoxic-ischemic encephalopathy were most frequently reported (4). Studying the local neonatal disease spectrum is important for the prevention and treatment of neonatal disease. However, to date, reports have mainly focused on the developed areas of China, such as Beijing (5), Hefei (6), Changsha (7), and Shanghai (8). These studies found that the types, order and mortality rate of diseases change over time, and reported that gestational age (9–13) and birth weight (14–16) are significantly correlated with neonatal diseases. As gestational age increases, the neonatal mortality (12), prevalence and length of hospital stay decreases (11). Low birth weight increases the prevalence of diseases (15).

Given the lack of reports on neonatal diseases in NICUs in China's underdeveloped areas, this study selected all newborns admitted to the NICU of a hospital in Xiangxi over the past 9 years. We retrospectively describe the basic conditions, disease spectrum and factors influencing newborns in the NICU.

## Materials and Methods

### Hospital Status

The Xiangxi area has 17.88 million people, accounting for 27.2% of the total population of Hunan Province of China. Although Xiangxi is the only underdeveloped area in Hunan, a large gap exists in the medical treatment level in this area compared with relatively developed areas. The NICU selected for this study is part of the largest hospital in the Xiangxi area with the highest standard of medical treatment in that region. It is a treatment center for critically-ill newborns, with 11 doctors (two professors and two associate professors) and 38 nursing staff (three associate professors). The NICU has 50 ordinary beds and 10 intensive care rescue beds. Medical equipment used in the unit includes neonatal ventilators, incubators, neonatal jaundice therapeutic devices, monitors, newborn hearing screeners, and hypothermia therapy apparatus, etc. The hospital uses electronic databases to record neonatal status, ensuring data is accurate and credible. The data collected in this hospital were considered to represent the disease spectrum and development of newborn diseases in the Xiangxi area.

From 2012 to 2020, there were 25,656 newborns born in this hospital, of which 9,615 (37.33%) were inborn (admitted to NICU after birth in this hospital) newborn. Table 1 shows that the total number of newborns born in this hospital reached a peak in 2016/2017 and then decreased year by year. This is likely related to China's “Two-child policy” to encourage larger families, introduced in 2015 (17). In addition, the neonatal mortality rate of Xiangxi in 2020 was 3.72‰, which was higher than that reported by WHO (18) in 2019 the Republic of Korea (2‰), Germany (2‰) and the United Kingdom (3‰), close to the China (4‰) and the United States (4‰), lower than South Africa (11‰), India (22‰) and world average (17.5‰). In recent years, China had established referral networks and regional treatment centers, which would reduce the neonatal mortality rate. Recently, China planned to reduce the neonatal mortality rate to 3.1‰ by 2025 (19).

**Table 1:** The total number of newborns born in one hospital in the Xiangxi area and the status of newborns admitted to the NICU.

**Year**	**Total number of newborns born in this hospital**	**Total number of newborns in NICU**	**Number of inborn**
2012	2,372	1,884	758
2013	2,368	1,975	1,201
2014	2,758	1,972	1,131
2015	2,749	1,822	1,056
2016	3,460	1,747	1,007
2017	3,968	1,782	1,143
2018	2,812	1,724	1,112
2019	2,853	1,730	1,207
2020	2,420	1,458	1,000
Total	25,728	16,094	9,615

### Study Population

**Figure 1:**
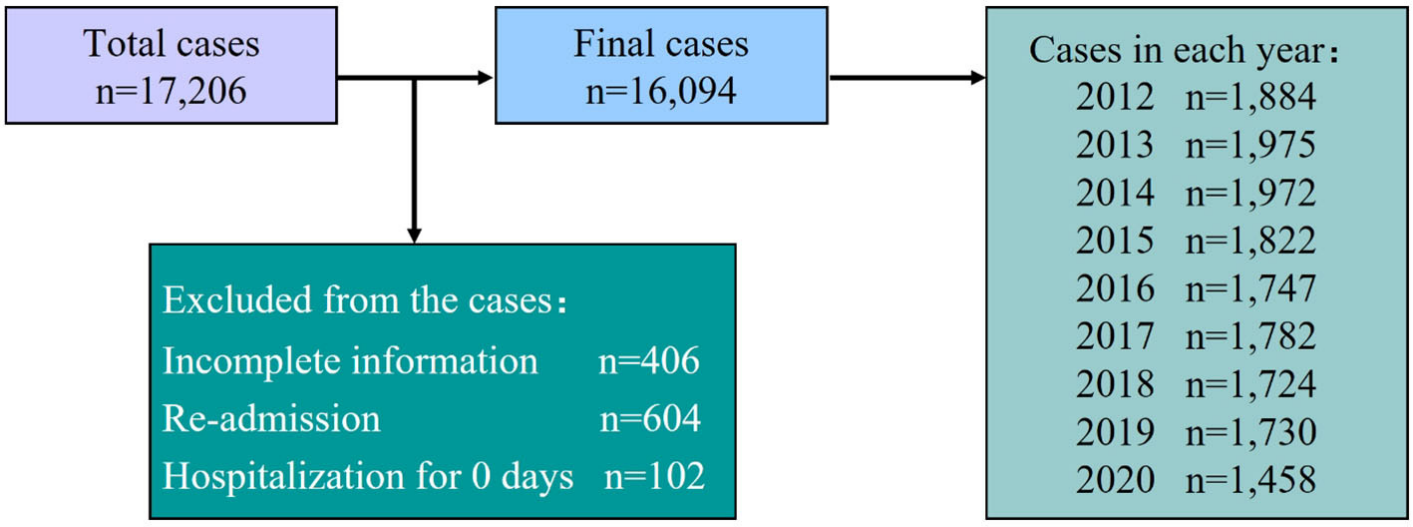
Overview of the study sample.

After approval by the Ethics Committee, data collection took place using the hospital database. All data were reviewed by two researchers. We undertook a retrospective analysis of all hospitalized newborns in the NICU of a hospital in Xiangxi from 2012 to 2020. A total of 17,206 newborns were initially included in this study. A total of 1,112 cases were excluded (406 had incomplete information, 604 had been re-admitted to the hospital, and 102 remained in hospital for less than a day). Finally, 16,094 newborns were included [of which 9,615 were inborn (admitted to NICU after birth in this hospital) newborn, and 6,479 were outborn (transferred from outside hospitals) newborn; see Figure 1].

### Methods

Statistical analysis of basic information about NICU newborns was undertaken, including hospitalization number, gender, gestational age, delivery method, birth weight, and patient source. This information was then related to discharge diagnosis, according to the International Statistical Classification of Diseases and Related Health Problems (10th Revision, ICD-10) breakdown and classification of children's diseases (see Table 2). Then exploratory data analysis was carried out based on neonatal disease diagnosis. The analysis was based on patients' first admission data, and any readmission situations were not considered for this part of the study.

**Table 2:** Disease classification and examples of diseases (ICD-10 codes).

**Disease classification**	**Disease (ICD-10 codes)**
Jaundice	Neonatal jaundice (P59), neonatal bilirubin encephalopathy (P57), etc.
Respiratory diseases	Neonatal pneumonia (P23), neonatal respiratory distress syndrome (P22), etc.
Neurological diseases	Hypoxic-ischemic encephalopathy (P91), intraventricular hemorrhage (P52), etc.
Cardiovascular diseases	Congenital heart disease (Q24), neonatal heart failure (P29), etc.
Blood system diseases	Neonatal anemia (P61), neonatal hemorrhagic disease (P53), etc.
Digestive system diseases	Neonatal gastrointestinal bleeding (P54), necrotizing enterocolitis (P77), etc.
Acid-base balance and electrolyte fouling	Electrolyte flocculation (E87), neonatal acidosis (P20), etc.
Infectious diseases	Neonatal purulent meningitis (G00), neonatal sepsis (P36), etc.
Asphyxia	Neonatal asphyxia (P21)
Birth injury diseases	Birth injury (P15), neonatal scalp hematoma caused by birth injury (P12), etc.
Eye, ear, throat, and nose diseases	Retinopathy of prematurity (H351), congenital laryngeal stridor (Q31), etc.
Skin diseases	Dermatitis (L23–26), eczema (L20), etc.
Endocrine and metabolic diseases	Neonatal hypoglycemia (P70), hyperparathyroidism (E21), etc.
Genitourinary system diseases	Renal insufficiency (N19), urinary tract infections in newborns (P39), etc.
Other diseases	Cold injury syndrome (P80), carbon monoxide poisoning (T58), etc.
Genetic diseases	Broad bean disease (D55), down syndrome (Q90), etc.
Musculoskeletal system diseases	Multi-finger (Q69), toe deformities (Q70), etc.
Immune diseases	Immunodeficiency (D84), etc.

The WHO classification of “Global causes of childhood deaths in 2010” (20) (Table 3) had been used to present the NICU disease spectrum in the Xiangxi area.

**Table 3:** Cause of neonatal death categories and ICD-10 codes.

**Disease classification**	**Disease (ICD-10 codes)**
Complications from preterm	P01.0, P01.1, P07, P22, P25-P28, P61.2, P77, etc.
Intrapartum related condition	P01.7-P02.1, P02.4-P02.6, P03, P10-P15, P20-P21, P24, P50, P90-P91
Infections	P35-P39 (excluding P37.3, P37.4)
Congenital abnormalities	Q00-Q99
Other causes	Remainder

### Statistical Analysis

We used SPSS 21.0 software for data sorting and analysis. The enumeration data are expressed by the constituent ratio or rate, measurement data by the mean ± standard deviation, and the difference between the two groups was analyzed by the chi-square and *t*-tests. A *P-*Value of <0.05 was considered statistically significant.

## Results

### Basic Description of Neonates Admitted to the NICU

#### Overall Situation

A total of 16,094 hospitalized newborns were included in this study, including 9,482 males and 6,612 females. The average male/female ratio was 1.43:1. Average gestational age was 37.14 ± 2.82 weeks (w), and the average birth weight was 2,803.34 ± 728.05 g. The total number of newborns and male/female ratio decreased each year, while the gestational age and birth weight remained stable from year to year (Table 4).

**Table 4:** Basic information about hospitalized newborns from 2012 to 2020.

**Year**	**Sample**	**Males/females**	**Ratio (males/females)**	**Gestational age (weeks)**	**Birth weight (g)**
2012	1,884	1,216/668	1.82	37.59 ± 2.73	2,865.63 ± 703.43
2013	1,975	1,189/786	1.51	36.42 ± 2.84	2,713.25 ± 690.71
2014	1,972	1,188/784	1.52	37.39 ± 2.61	2,822.80 ± 732.62
2015	1,822	1,065/757	1.41	36.58 ± 2.76	2,627.60 ± 728.57
2016	1,747	990/757	1.31	37.31 ± 2.82	2,855.26 ± 710.05
2017	1,782	1,026/756	1.36	36.74 ± 2.82	2,769.79 ± 745.30
2018	1,724	982/742	1.32	37.41 ± 2.71	2,847.94 ± 736.07
2019	1,730	999/731	1.37	37.38 ± 2.97	2,897.31 ± 729.64
2020	1,458	827/631	1.31	37.34 ± 3.10	2,852.72 ± 740.66
Total	16,094	9,482/6,612	1.43	37.14 ± 2.82	2,803.34 ± 728.05

#### Delivery Method

**Figure 2:**
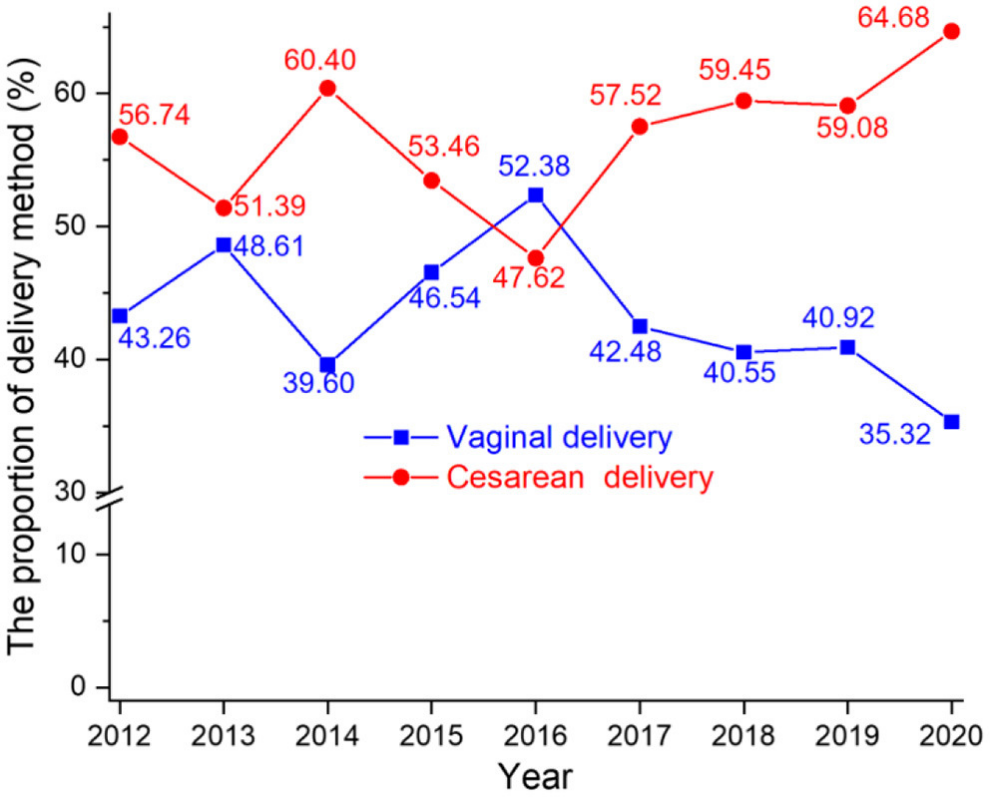
The proportion of vaginal delivery and cesarean delivery from 2012 to 2020.

From 2012 to 2020, two delivery methods were recorded: vaginal delivery (VD) and cesarean delivery (CD). A total of 6,965 (43.48%) of infants were delivered naturally (VD); 9,096 (56.52%) *via* CD. In 2012, the proportions of VD and CD were 43.26 and 56.74%, respectively. By 2020, the proportions had changed to 35.32 and 64.68%, respectively. Notably, the proportion of CD increased by 7.94% in 9 years. Figure 2 summarizes the changes in delivery methods over the study period.

#### Gestational Age

**Figure 3:**
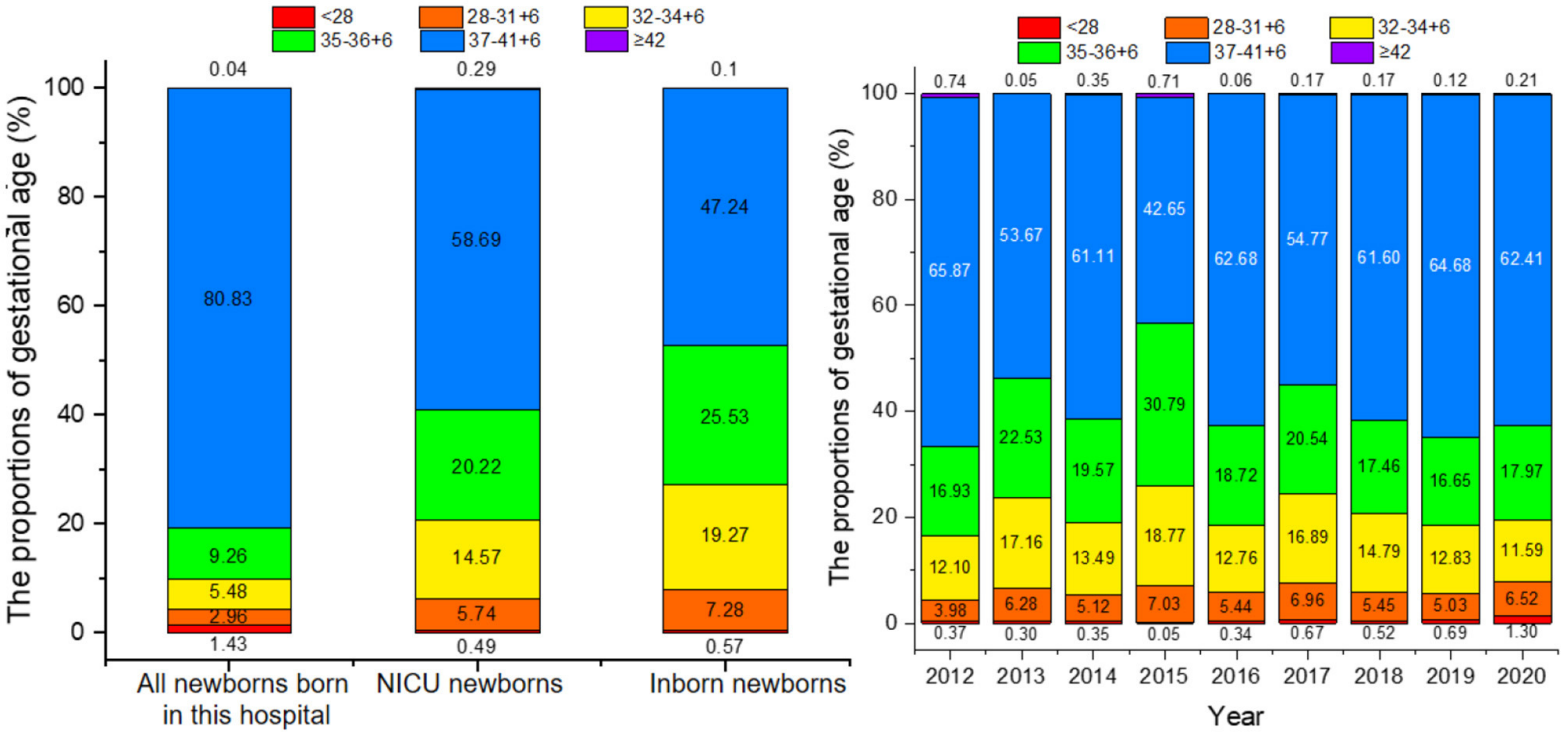
Gestational age distribution of newborns (left) and the proportion of gestational age of newborns from 2012 to 2020 (right).

Grouped the Xiangxi and inborn newborns of gestation to discuss referral preferences for newborns (see Figure 3, left). The results showed that the proportions of preterm infants (<37 weeks) were 19.13 and 52.66%, respectively. While the proportions of late-preterm infants (35–36+6 weeks) among preterm infants in the Xiangxi and inborn newborns were 48.41% (9.26%/19.13%) and 48.48% (25.53%/52.66%); the proportions of <28 weeks infants among preterm infants were 7.48% (1.43%/19.13%) and 1.08% (0.57%/52.66%), respectively. It indicated that there was no obvious referral preference for late-preterm infants, and occurred referral preference for <28 weeks infants.

In the 9-year period, the minimum and maximum gestational age of NICU newborns was 23 and 43+1 weeks. After grouping infants by gestational age, we found that the NICU housed mainly full-term infants, with a sample of 9,445 accounting for 58.69% of the total sample. Preterm infants comprised 41.02% of the sample (*n* = 6,602), which mainly included 35–36+6 weeks late-preterm infants. The highest proportion of preterm infants was noted in 2015 with 56.64% of the sample that year, 19.26% higher than in 2020 (see Figure 3, right).

#### Birth Weight

**Figure 4:**
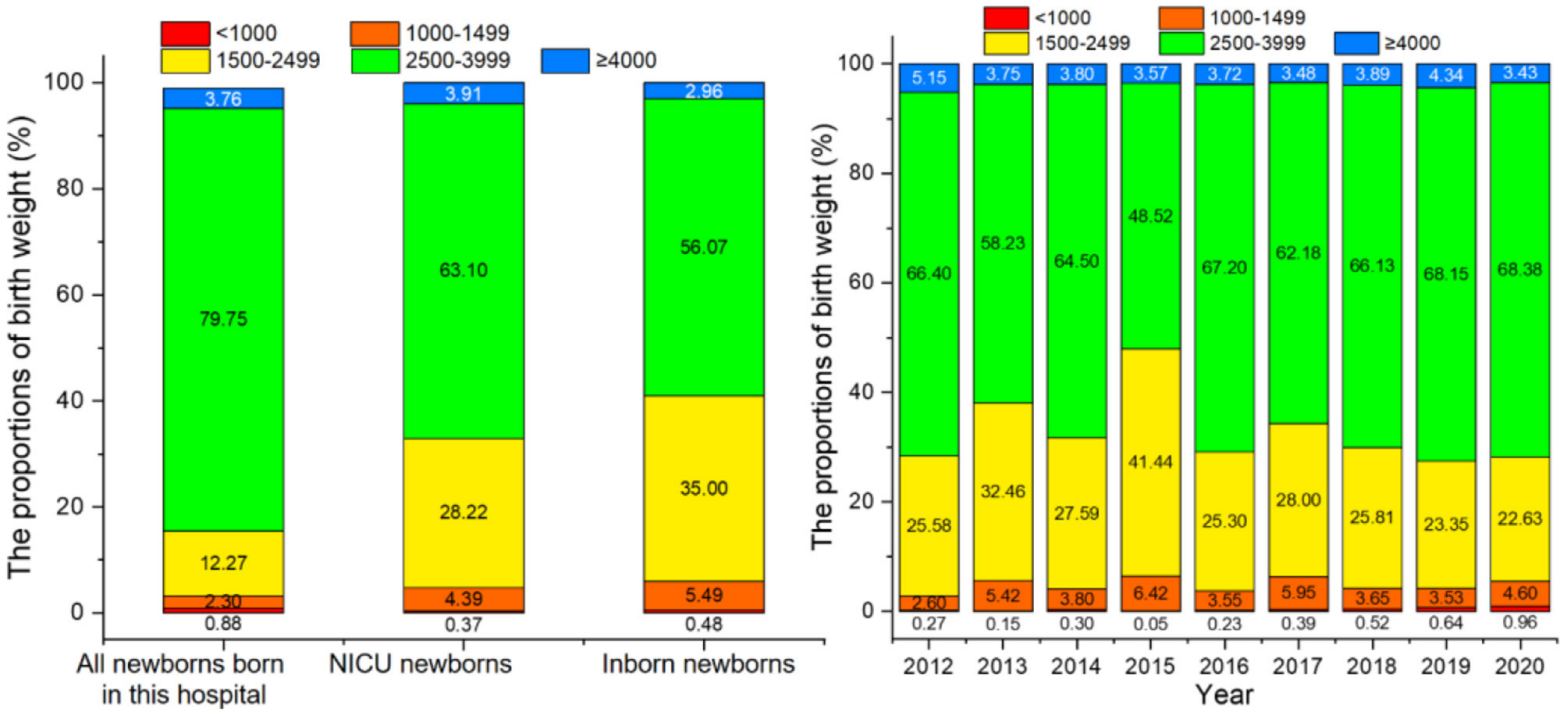
The birth weight distribution of newborns (left) and the birth weight of newborns from 2012 to 2020 (right).

Comparing all newborns born in this hospital, NICU newborns and inborn newborns, it was found that the proportions of low birth weight infants (<2,500 g) were 15.45, 32.99, and 40.97%, respectively (see Figure 4, left).

The minimum and maximum birth weight of hospitalized newborns was 330 and 5,530 g. Birth weight was divided into five groups of <1,000, 1,000–1,499, 1,500–2,499, 2,500–3,999, and ≥4,000 g, with the average proportion for each group being 0.37, 4.39, 28.22, 63.10, and 3.91%, respectively. Normal weight infants accounted for most of the newborns (63.10%), followed by low birth weight infants (<2,500 g) at 28.22%. The highest proportion of low birth weight infants was seen in 2015, consistent with the highest proportion of preterm infants in that same year (Figure 4, right).

#### Patient Source

**Figure 5:**
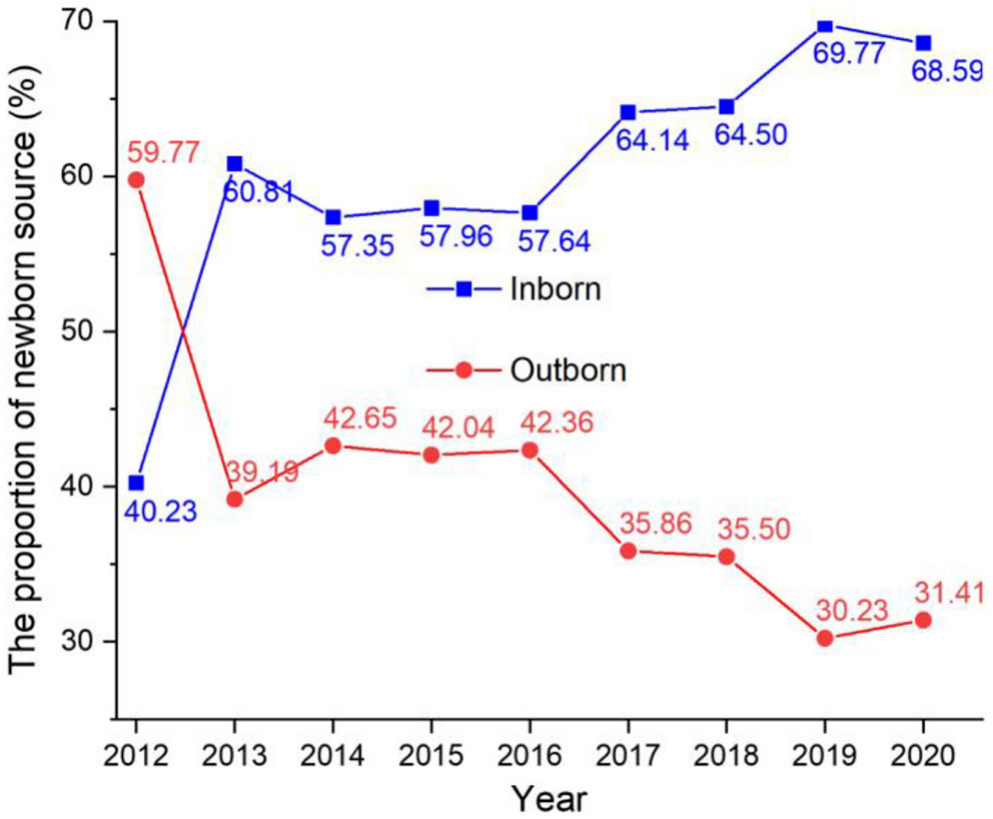
The proportion of newborns by source (inborn/outborn) from 2012 to 2020.

In the 9 year period of our study, 9,615 (59.74%) hospitalized newborns were inborn, and 6,479 (40.26%) were outborn (see Figure 5). The proportion of outborn newborns declined during the study period, from 59.77% in 2012 to 31.41% in 2020. The proportion of inborn newborns increased in the study period from 40.23% in 2012 to 68.59% in 2020.

### Neonatal Disease

#### Disease Spectrum

**Figure 6:**
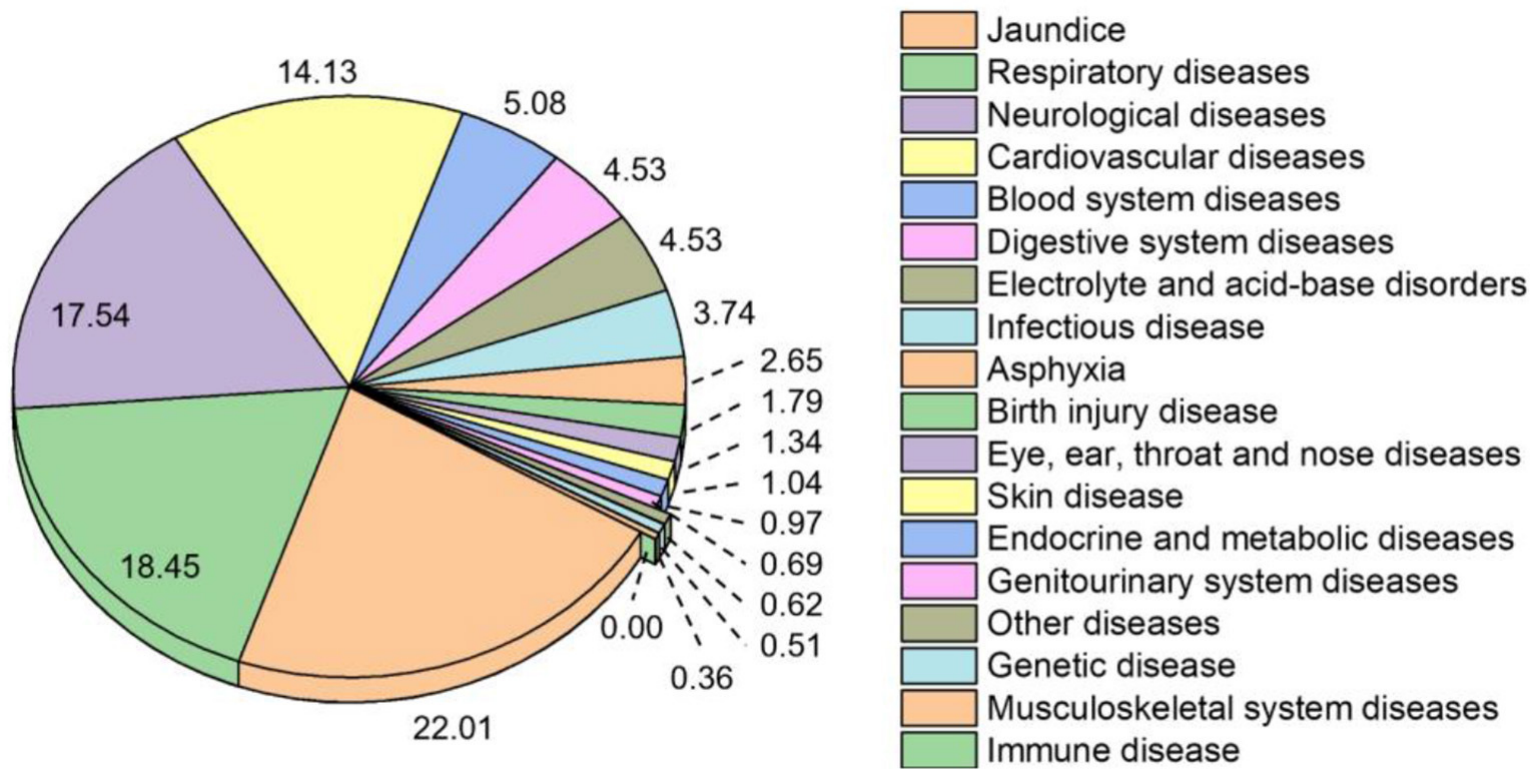
Disease spectrum of infants in the NICU over the study period *via* ICD-10. Note: The prevalence of neonatal disease is expressed as a percentage (%) of all reported diseases.

Based on the discharge diagnosis of all hospitalized newborns, Figure 6 shows the total disease spectrum of infants in the NICU over the past 9 years. The most prevalent neonatal disease was jaundice (22.01%), followed by respiratory diseases (18.45%), neurological diseases (17.54%), and cardiovascular diseases (14.13%).

**Figure 7:**
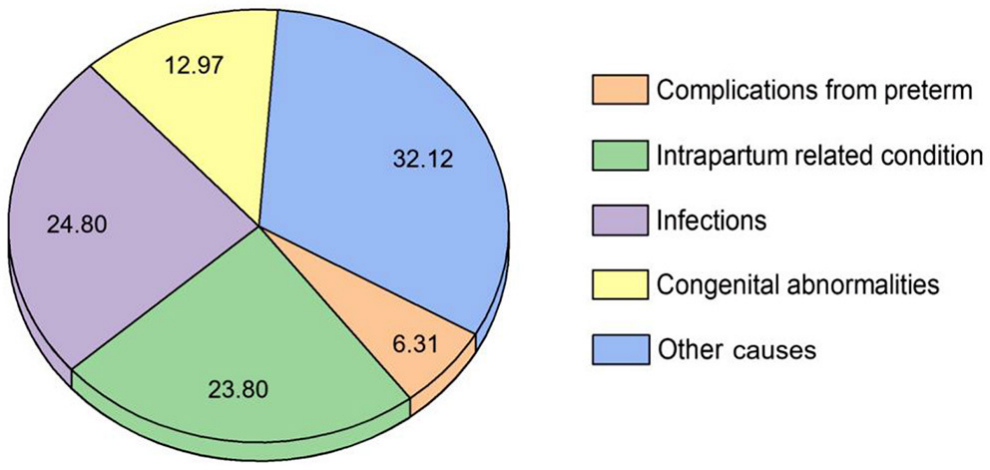
Disease spectrum of infants in the NICU over the study period *via* WHO classification. Note: The prevalence of neonatal disease is expressed as a percentage (%) of all reported diseases.

The WHO classification of “Global causes of childhood deaths in 2010” (20) has been used to present the NICU disease spectrum in the Xiangxi area. As show in the Figure 7, the most prevalent neonatal disease was other causes (32.12%), followed by infections (24.80%), intrapartum related condition (23.80%), congenital abnormalities (12.97%), and complications from preterm (6.31%).

#### Changes in Neonatal Diseases by Year

The prevalence of neonatal diseases from 2012 to 2020 is summarized in Table 5. Jaundice and cardiovascular disease prevalence increased, while occurrence of respiratory and neurological diseases decreased. The prevalence of the remaining diseases remained almost unchanged. Interestingly, the prevalence of jaundice increased each year from 2012. In 2012, jaundice ranked as the third most prevalent condition, but it had moved to be most common in 2015–2020.

**Table 5:** The prevalence of neonatal diseases from 2012 to 2020.

**Disease**	**2012**	**2013**	**2014**	**2015**	**2016**	**2017**	**2018**	**2019**	**2020**
Jaundice	18.17	21.57	20.53	20.13	23.41	20.02	24.09	26.48	27.07
Respiratory diseases	25.57	18.98	16.57	16.39	19.58	15.40	19.01	20.13	19.55
Neurological diseases	21.84	18.13	22.28	17.97	23.22	19.92	13.89	7.50	1.92
Cardiovascular diseases	6.83	13.77	11.89	15.21	13.93	15.41	14.19	16.40	22.57
Blood system diseases	4.44	4.72	4.69	5.10	4.02	5.91	5.52	5.67	5.77
Digestive system diseases	4.31	4.59	4.57	4.80	4.21	4.65	4.92	4.51	4.17
Acid-base balance and electrolyte fouling	3.87	3.92	4.48	5.02	2.85	6.67	5.08	4.91	4.02
Infectious diseases	3.60	4.13	5.85	4.34	2.41	2.10	3.37	4.35	3.40
Asphyxia	3.23	3.12	2.58	3.18	2.07	2.31	2.43	1.92	2.18
Birth injury diseases	2.54	2.14	1.59	2.32	0.80	1.49	1.83	1.68	1.68
Eye, ear, throat, and nose diseases	1.13	1.09	0.73	0.98	0.67	2.79	1.73	1.58	1.61
Skin diseases	0.98	1.12	1.20	1.09	0.80	0.89	0.94	1.12	1.29
Endocrine and metabolic diseases	0.91	0.91	0.62	1.04	0.34	0.44	0.92	1.66	2.56
Genitourinary system diseases	0.81	0.53	0.56	0.52	0.63	0.76	0.92	0.88	0.81
Other diseases	0.76	0.62	0.81	1.16	0.19	0.37	0.44	0.38	0.26
Genetic diseases	0.68	0.34	0.70	0.27	0.56	0.52	0.38	0.48	0.55
Musculoskeletal system diseases	0.34	0.32	0.34	0.47	0.29	0.33	0.36	0.32	0.57
Immune diseases	0.00	0.00	0.00	0.00	0.00	0.03	0.00	0.00	0.00

*Prevalence is expressed as a percentage (%) of the total study sample*.

### Influencing Factors

The chi-square test was used to compare the prevalence of neonatal diseases with different characteristics. The prevalence of neonatal diseases by patient source, gender, delivery method, gestational age, and birth weight were shown to be statistically significant (*P* < 0.05) (Table 6).

**Table 6:** Prevalence of neonatal diseases in relation to different characteristics of the sample.

**Characteristic**		**Number of newborns**	**Number of cases of neonatal diseases**	**χ^2^**	***P*-Value**
Delivery method	Vaginal delivery	6,998	6,976	21.83	<0.01*
	Cesarean delivery	9,096	9,013		
Gender	Male	9,482	9,419	491.50	<0.01*
	Female	6,612	6,570		
Gestational age (weeks)	<37	6,602	6,512	43.867	<0.01*
	37–41+6	9,445	9,430		
	≥42	47	47		
Birth weight (g)	<2,500	5,309	5,241	24.207	<0.01*
	2,500–3,999	10,155	10,120		
	≥4,000	630	628		
Patient source	Inborn	9,615	9,528	23.478	<0.01*
	Outborn	6,479	6,461		

*Indicates statistically significant (P <0.05).

#### Delivery Method

**Figure 8:**
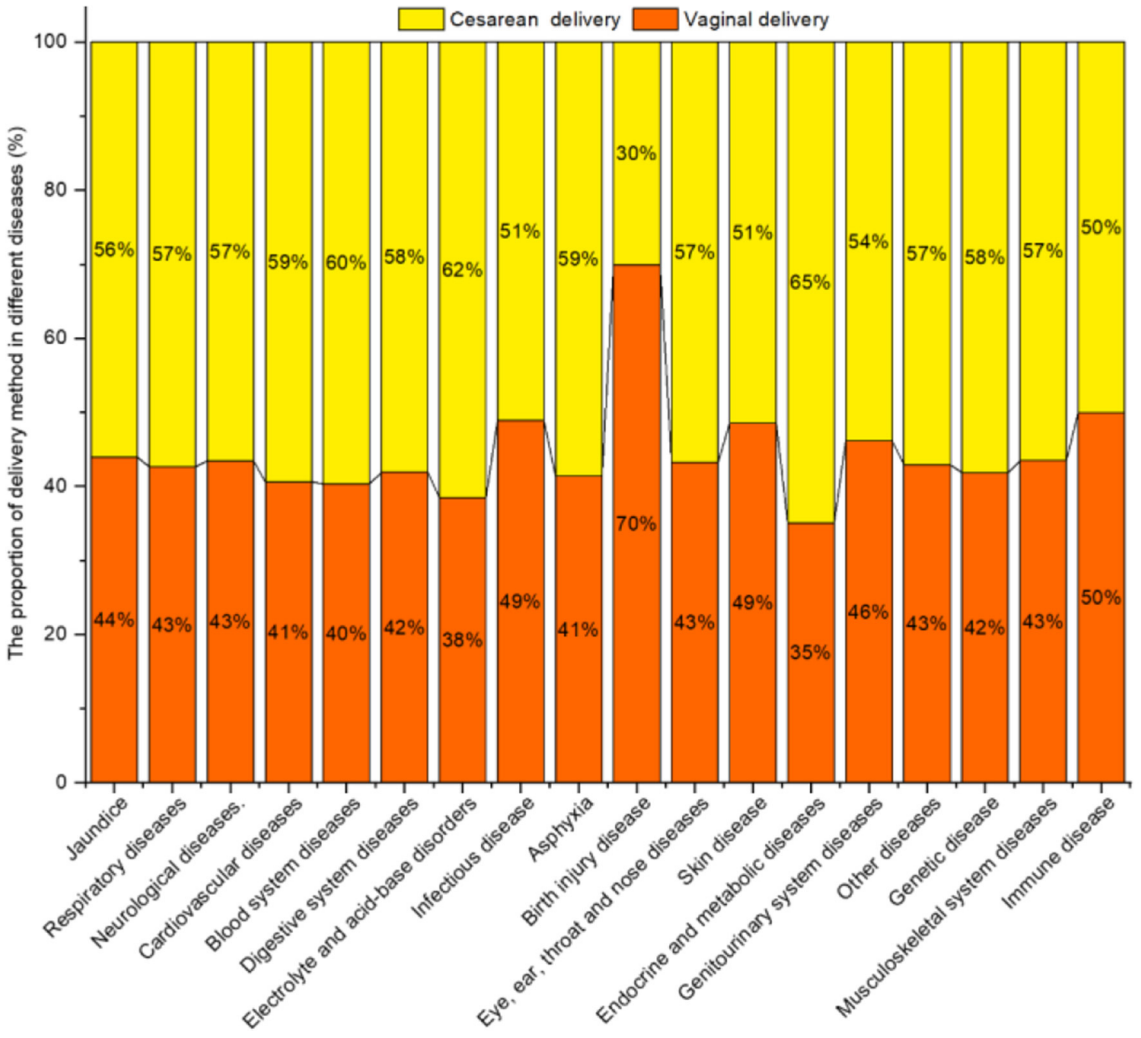
Neonatal diseases and distribution by delivery method.

Figure 8 shows the proportion of delivery method for diseases. Except for Birth injury diseases, the prevalence of jaundice, respiratory diseases, neurological diseases, cardiovascular diseases, blood system diseases, digestive system diseases, electrolyte and acid–base disorders, and asphyxia, etc. in infants born by CD was higher than for VD.

The chi-square test showed that the prevalence of respiratory diseases, cardiovascular diseases, blood system diseases, electrolyte and acid-base disorders, infectious disease, birth injury disease, skin disease, endocrine, and metabolic diseases differed significantly for different delivery methods (*P* < 0.05) (Table 7).

**Table 7:** Prevalence of neonatal diseases in relation to delivery method.

**Disease**	**Vaginal delivery**	**Cesarean delivery**	**χ^2^**	***P*-Value**
	***n* = 6,998**	***n* = 9,096**		
Jaundice	4,891	6,238	3.19	>0.05
Respiratory diseases	3,978	5,352	6.46	<0.05*
Neurological diseases	3,854	5,018	0.014	>0.05
Cardiovascular diseases	2,902	4,246	43.50	<0.01*
Blood system diseases	1,036	1,531	12.13	<0.01*
Digestive system diseases	961	1,332	2.67	>0.05
Electrolyte and acid-base disorders	881	1,410	27.47	<0.01*
Infectious diseases	924	965	25.70	<0.01*
Asphyxia	555	787	2.69	>0.05
Birth injury diseases	633	272	273.25	<0.01*
Eye, ear, throat, and nose diseases	294	386	0.018	>0.05
Skin diseases	256	271	5.75	<0.05*
Endocrine and metabolic diseases	173	320	14.57	<0.01*
Genitourinary system diseases	161	188	1.02	>0.05
Other diseases	134	178	0.04	>0.05
Genetic diseases	108	150	0.28	>0.05
Musculoskeletal system diseases	80	104	0	>0.05
Immune diseases	1	1	0.04	>0.05

*Indicates statistically significant (P <0.05). Each patient is counted for multiple diseases, not only the single main diagnosis.

#### Gender

**Figure 9:**
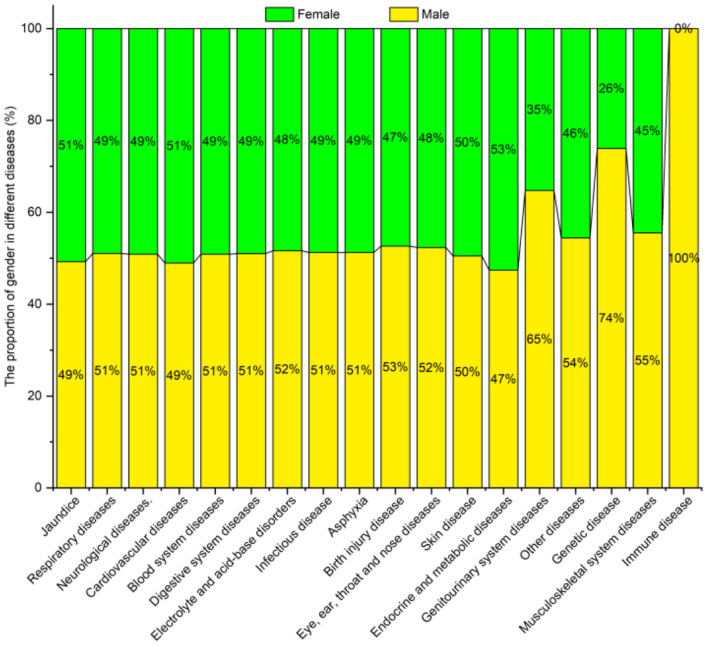
Neonatal diseases and distribution by gender.

Figure 9 shows the diseases and their proportionate spread by gender. The prevalence of most diseases was higher in males than in females, e.g., for respiratory, neurological, blood system, and digestive system diseases. Females only had a higher prevalence of jaundice, cardiovascular, endocrine, and metabolic diseases than males.

The chi-square test indicated a significant difference (*P* < 0.05) in the prevalence of jaundice, respiratory, neurological, cardiovascular, genitourinary system, and genetic diseases among the groups, while the prevalence of blood system diseases, digestive system diseases, electrolyte and acid-base disorders and other diseases were not statistically different for the gender (*P* > 0.05) (see Table 8).

**Table 8:** Prevalence of neonatal diseases in relation to gender.

**Disease**	**Male**	**Female**	**χ^2^**	***P*-Value**
	***n* = 9,482**	***n* = 6,612**		
Jaundice	6,475	4,654	8.05	<0.01*
Respiratory diseases	5,592	3,738	9.53	<0.01*
Neurological diseases	5,301	3,571	5.67	<0.05*
Cardiovascular diseases	4,139	3,009	5.44	<0.05*
Blood system diseases	1,534	1,033	0.89	>0.05
Digestive system diseases	1,373	920	1.02	>0.05
Electrolyte and acid-base disorders	1,386	905	2.76	>0.05
Infectious diseases	1,136	753	1.32	>0.05
Asphyxia	807	535	0.9	>0.05
Birth injury diseases	556	349	2.52	>0.05
Eye, ear, throat, and nose diseases	416	264	1.5	>0.05
Skin diseases	313	214	0.05	>0.05
Endocrine and metabolic diseases	278	215	1.34	>0.05
Genitourinary system diseases	253	96	27.17	<0.01*
Other diseases	197	115	2.35	>0.05
Genetic diseases	207	51	49.22	<0.01*
Musculoskeletal system diseases	118	66	2.09	>0.05
Immune diseases	2	0	1.39	>0.05

*Indicates statistically significant (P <0.05). Each patient is counted for multiple diseases, not only the single main diagnosis.

#### Gestational Age

**Figure 10:**
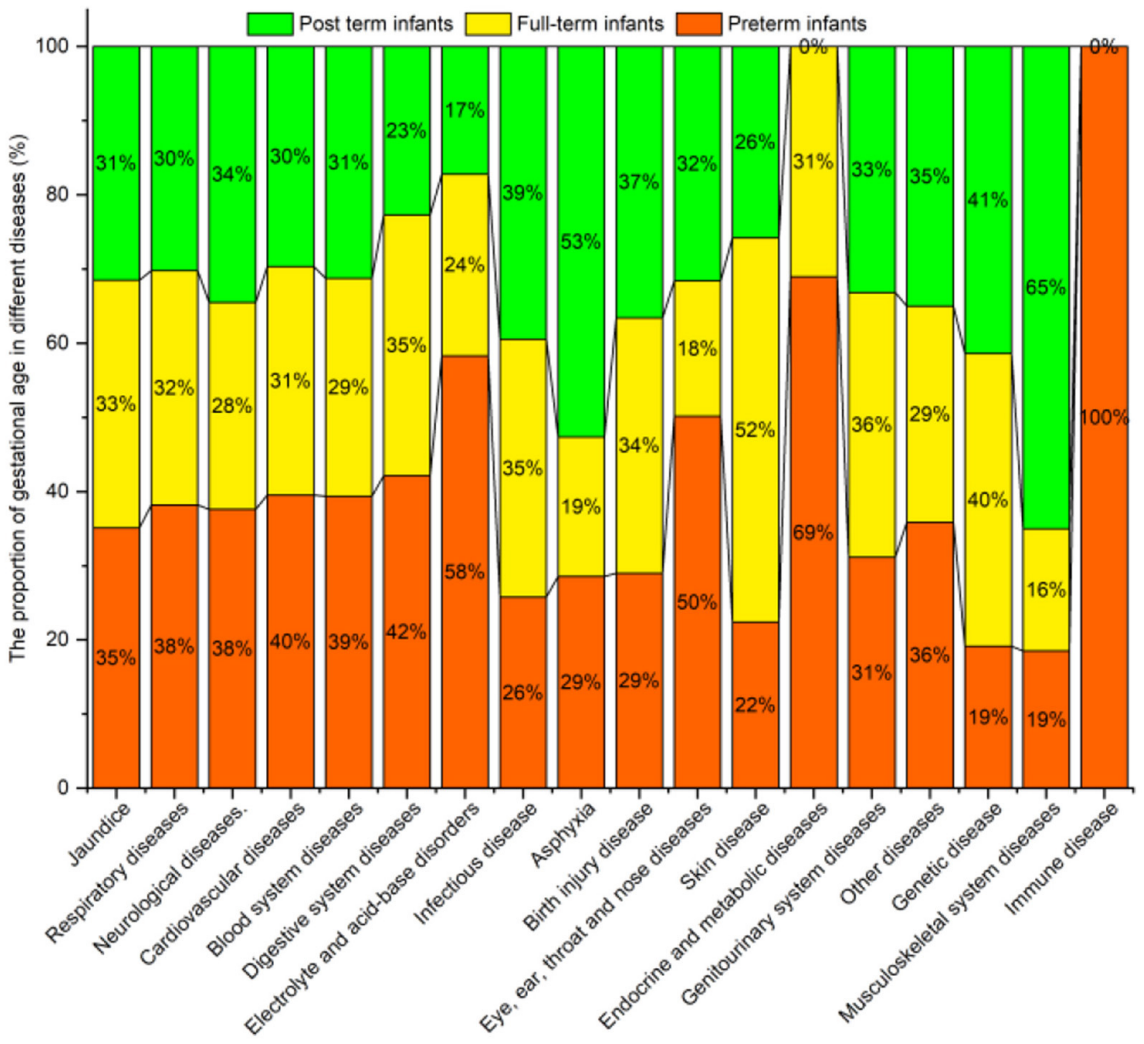
Neonatal diseases and distribution by gestational age.

Figure 10 shows the prevalence of neonatal diseases for infants born at different gestational ages. Prevalence of most neonatal diseases changed with an increase in gestational age, such as the prevalence of jaundice, respiratory, neurological, cardiovascular, blood system, digestive system, and electrolyte and acid-base disorders, which gradually decreased with increasing gestational age. In contrast, the prevalence of infectious disease, asphyxia, and birth injury gradually increase with gestational age.

Table 9 shows the prevalence of neonatal diseases in relation to gestational age. No statistical difference was found between the gestational age groups for genitourinary system, musculoskeletal system, immune, and other diseases (*P* > 0.05). However, a significant difference between the gestational age groups (*P* < 0.05) was found for jaundice, asphyxia, respiratory, neurological, cardiovascular, blood system, digestive system, electrolyte and acid-base, infectious, birth injury, eye, ear, throat and nose, skin, endocrine and metabolic, and genetic diseases.

**Table 9:** Prevalence of neonatal diseases in relation to gestational age.

**Disease**	** <37 weeks**	**37–41+6 weeks**	**≥42 weeks**	**χ^2^**	***P*-Value**
	***n* = 6,602**	***n* = 9,445**	***n* = 47**		
Jaundice	4,702	6,397	30	22.84	<0.01*
Respiratory diseases	4,261	5,045	24	733.79	<0.01*
Neurological diseases	4,289	4,555	28	440.50	<0.01*
Cardiovascular diseases	3,372	3,758	18	201.24	<0.01*
Blood system diseases	1,238	1,322	7	65.58	<0.01*
Digestive system diseases	1,044	1,245	4	23.30	<0.01*
Electrolyte and acid-base disorders	1,429	859	3	503.70	<0.01*
Infectious diseases	642	1,240	7	43.92	<0.01*
Asphyxia	686	647	9	70.94	<0.01*
Birth injury diseases	334	568	3	6.72	<0.05*
Eye, ear, throat, and nose diseases	446	232	2	177.49	<0.01*
Skin diseases	122	404	1	72.61	<0.01*
Endocrine and metabolic diseases	300	193	0	83.32	<0.01*
Genitourinary system diseases	132	216	1	1.51	>0.05
Other diseases	144	167	1	3.50	>0.05
Genetic diseases	65	192	1	27.15	<0.01*
Musculoskeletal system diseases	80	102	2	4.64	>0.05
Immune diseases	2	0	0	2.88	>0.05

*Indicates statistically significant (P <0.05). Each patient is counted for multiple diseases, not only the single main diagnosis.

#### Birth Weight

**Figure 11:**
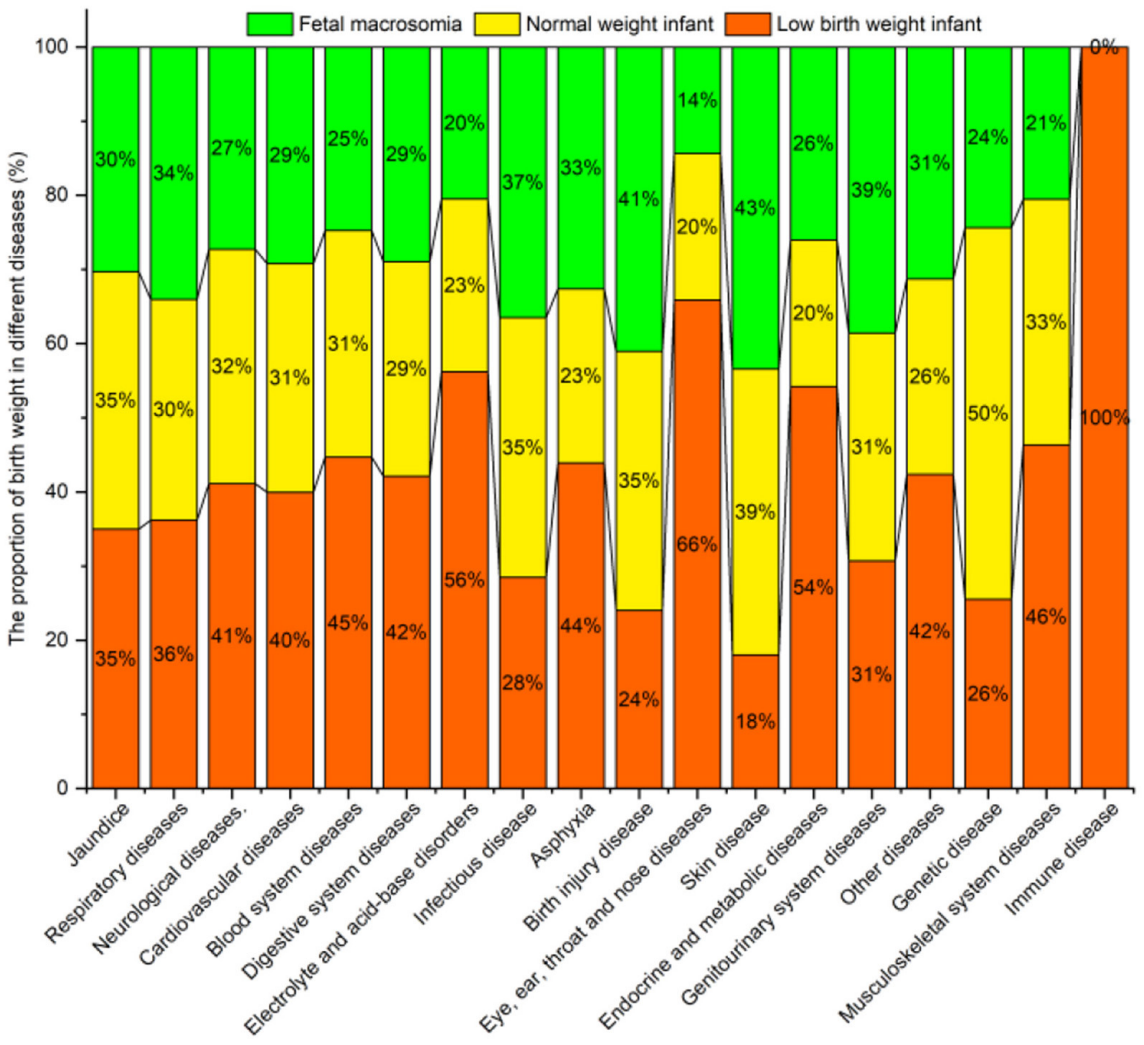
Neonatal diseases and distribution by birth weight.

Figure 11 shows the proportion of birth weight for diseases. The prevalence of most diseases in low birth weight infants was higher than in normal-weight infants and fetal macrosomia, e.g., for jaundice, neurological, cardiovascular, blood system, digestive system and electrolyte, and acid-base disorders. Further analysis found that the prevalence of respiratory diseases and asphyxia in infants with excessive/low birth weight was higher than that of normal-weight infants.

No significant difference (*P* > 0.05) was found between the birth weight groups for genitourinary system diseases. However, significant differences were found between the groups for all the remaining diseases (Table 10).

**Table 10:** Prevalence of neonatal diseases in relation to birth weight.

**Disease**	** <2,500 g**	**2,500–3 999 g**	**≥4,000 g**	**χ^2^**	***P*-Value**
	***n* = 5,309**	***n* = 10,155**	***n* = 630**		
Jaundice	3,713	7,034	382	23.20	<0.01*
Respiratory diseases	3,478	5,464	388	199.58	<0.01*
Neurological diseases	3,481	5,117	274	360.59	<0.01*
Cardiovascular diseases	2,790	4,116	242	213.61	<0.01*
Blood system diseases	1,081	1,415	71	118.15	<0.01*
Digestive system diseases	957	1,258	78	92.58	<0.01*
Electrolyte and acid-base disorders	1,248	989	54	558.55	<0.01*
Infectious diseases	539	1,268	82	19.37	<0.01*
Asphyxia	636	650	56	142.23	<0.01*
Birth injury diseases	227	632	46	28.40	<0.01*
Eye, ear, throat, and nose diseases	425	244	11	280.38	<0.01*
Skin diseases	98	401	28	51.51	<0.01*
Endocrine and metabolic diseases	281	196	16	133.38	<0.01*
Genitourinary system diseases	114	218	17	0.87	>0.05
Other diseases	137	163	12	17.45	<0.01*
Genetic diseases	53	199	6	22.19	<0.01*
Musculoskeletal system diseases	76	104	4	6.62	<0.05*
Immune diseases	2	0	0	4.06	<0.01*

*Indicates statistically significant (P <0.05). Each patient is counted for multiple diseases, not only the single main diagnosis.

#### Patient Source

**Figure 12:**
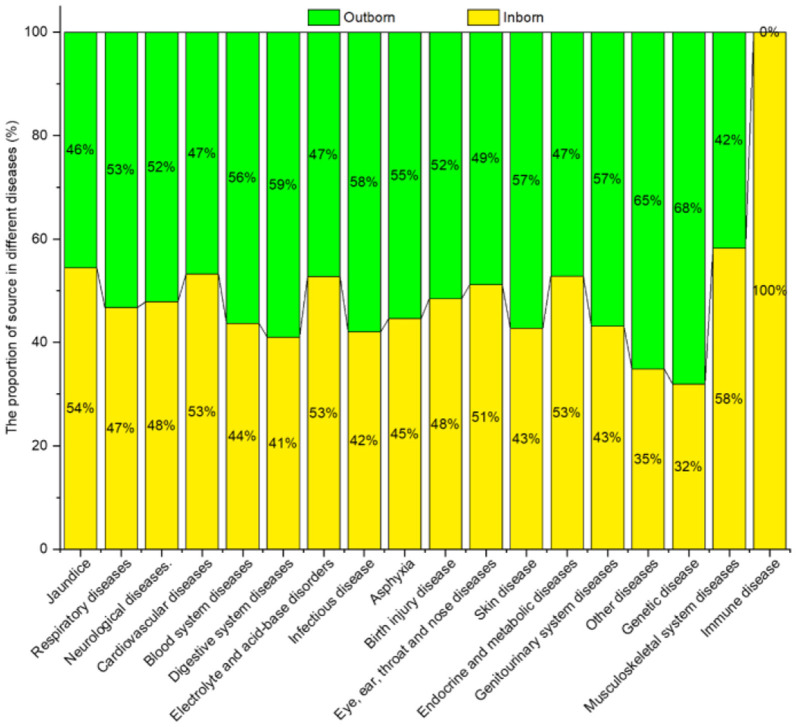
Neonatal diseases and distribution by patient source.

Figure 12 shows the neonatal diseases and their distribution in relation to patient source. Outborn newborns were mainly affected by respiratory diseases, neurological diseases, blood system diseases, digestive system diseases, and infectious diseases. While inborn newborns were primarily diagnosed with jaundice.

No significant difference in the prevalence of birth injury disease, eye, ear, throat and nose diseases, endocrine and metabolic diseases, and immune diseases were found among the groups (*P* > 0.05). However, significant differences were noted between groups (*P* < 0.05) for all the remaining diseases, as shown in Table 11.

**Table 11:** Prevalence of neonatal diseases in relation to patient source.

**Disease**	**Inborn**	**Outborn**	**χ^2^**	***P*-Value**
	***n* = 9,615**	***n* = 6,479**		
Jaundice	7,121	4,008	270.06	<0.01*
Respiratory diseases	5,280	4,050	91.65	<0.01*
Neurological diseases	5,114	3,758	36.28	<0.01*
Cardiovascular diseases	4,489	2,659	50.00	<0.01*
Blood system diseases	1,374	1,193	49.09	<0.01*
Digestive system diseases	1,165	1,128	88.78	<0.01*
Electrolyte and acid-base disorders	1,428	863	7.44	<0.01*
Infectious diseases	979	910	55.77	<0.01*
Asphyxia	731	611	16.92	<0.01*
Birth injury diseases	527	378	0.91	>0.05
Eye, ear, throat, and nose diseases	414	266	0.38	>0.05
Skin diseases	277	250	11.68	<0.01*
Endocrine and metabolic diseases	308	185	1.58	>0.05
Genitourinary system diseases	185	164	6.73	<0.01*
Other diseases	138	174	31.83	<0.01*
Genetic diseases	106	152	37.95	<0.01*
Musculoskeletal system diseases	124	60	4.53	<0.05*
Immune diseases	2	0	1.35	>0.05

*Indicates statistically significant (P <0.05). Each patient is counted for multiple diseases, not only the single main diagnosis.

## Discussion

This study analyzed data from 16,094 newborn infants admitted to one NICU in 9 years. The average NICU male-to-female ratios were 1.43:1. Among them, the male-to-female ratios of the inborn and outborn newborns were 1.32:1 and 1.63:1, which were higher than the birth male-to-female ratios in China (1.12:1) and the United States (1.05:1) in 2019 (21). As shown in Table 12, grouped the male and female newborns of gestation to discuss referral preferences for gender. The results showed that the proportion of males and females in each gestational age group was similar in inborn/outborn newborns. It was worth noting that gender had significant effect on neonatal prevalence, with males had higher prevalence than females (22–24). This demonstrates that although males accounted for more resources, there was no gender bias in treatment and referral.

**Table 12:** The proportion (%) of male and female of inborn/outborn newborns in each gestational age (weeks).

**Characteristic**		** <37 weeks**	**37–41+6 weeks**	**>42 weeks**
Inborn	Male	2,925 (53.51)	2,535 (46.38)	6 (0.11)
	Female	2,138 (51.53)	2,007 (48.37)	4 (0.10)
Outborn	Male	963 (23.98)	3,029 (75.42)	24 (0.60)
	Female	576 (23.39)	1,874 (76.09)	13 (0.53)

Investigation of the relationship between maternal and neonatal characteristics was beneficial for improving perinatal health care. It could be found that the total number of newborn born in this hospital increased year by year from 2016 in Xiangxi. It was due to the policy implementation of China's “universal two-child policy” in 2016 (25). This policy significantly increased the proportion of advanced maternal age, maternal complications, and multiple pregnancies (26, 27). Moreover, the gestational age was attributed to the mother and newborn, which had significant impact on the neonatal disease. It was reported that >50% of full-term newborns occurred jaundice, which was more severe in late preterm infants (28, 29). In this work, the average proportion of premature infants was 41.02%, which was higher than the Chinese average of 26.2% (4). The prevalence of most neonatal diseases in infants in the NICU in Xiangxi significantly increased with decreased gestational age, especially jaundice. In addition, the delivery method of pregnancy had significant impact on neonatal disease (30). The prevalence of CD was significantly higher than that of VD (31). Linn (32) reported that high CD ratio increased the occurred of jaundice. The Lancet (33) reported that the safety of cesarean delivery had improved over the past 30 years with advances in perinatal medicine, resulting in an increase in CD rates worldwide. The WHO reported on The Lancet that China had one of the highest CD rates in the world, with the CD rate was 46.2% in 2008 (34). It was reported that the CD rates in China's developed cities had been decreasing over the past decade, while the CD rates in undeveloped regions had been increasing (35). In this work, the CD accounts for 48.60 and 56.52% of Xiangxi and NICU newborns, which were higher than the Chinese average in 2018 (36.7%) (33, 35). The characteristics of pregnancy and newborn in advanced maternal age (36), high CD rates (37), and premature infants (28, 29) increased the prevalence of jaundice. In conclusion, pregnancy characteristics were associated with the occurrence of newborn diseases, which required more attention and in-depth exploration.

The United Nations reported a global rate of 14.6% for low birth weight infants, most of whom are from Asia and Africa (38). However, in the past 9 years in the NICU in Xiangxi that formed the focus of this study, the prevalence of low birth weight infants was 28.22%, substantially higher than the 14.6% global prevalence of low birth weight infants in 2015 (39). The average weight of NICU newborns in our study was 2,803.34 ± 728.05 g, with low birth weight infants constituting 28.22%, very low birth weight infants 4.39% and extremely low birth weight infants 0.37%. These findings are consistent with reports that the birth weight of premature infants is mainly 1,500–2,500 g in China (7, 8).

Analysis of the disease spectrum in the NICU showed that the most common neonatal diseases are jaundice, respiratory diseases, and neurological diseases, consistent with Wei's (4) Chinese study. Our study found that the type and prevalence of neonatal diseases are closely related to gender, gestational age, patient sources, delivery methods, and birth weight. The prevalence of most diseases in males was higher than in females (22–24). The analysis indicated a statistically significant difference between the patient sources. Outborn infants had mainly been diagnosed with respiratory, neurological, blood system, digestive system, electrolyte, and acid-base disorders. We found the prevalence of most neonatal diseases in preterm and low birth weight infants was significantly higher than the term infants, post-term infants, normal weight infants and those with fetal macrosomia, consistent with recent reports (9–16).

China's medical treatment level was lower than that of developed countries. It was worth noting that the medical treatment level was more scarce in undeveloped areas. The lowest weight newborn successfully cured could represent the medical treatment level. The PICC technology was first introduced in the Xiangxi area in 2013, and successfully cured one 24 weeks and 650 g newborn in 2019. Which was higher than Gansu (24 weeks, 370 g) and Changsha (25+5 weeks, 400 g) of China, and the United States (23+3 weeks, 245 g) (40). In recent years, the referral network enabled newborns in Xiangxi who were difficult to cure to be referred to developed cities such as Changsha.

At present, China had proposed the “three-child policy,” and it was predicted that the proportion of advanced maternal age, the CD rates and preterm infants rate would increase. Therefore, some advice on pregnancy health care had been provided basis on the above study. Encourage fertility during women of childbearing age, to reduce the advanced maternal age and maternal complications. Increase the full-term delivery rate, and reduce the premature delivery rate. Encourage using the vaginal delivery method to reduce the CD rate, especially in undeveloped areas. Improving perinatal health care could effectively improve pregnancy outcomes and neonatal outcomes.

## Conclusions

The past 9 years have seen a decrease in the number of newborns, preterm infants and infants transferring from outside hospitals into the NICU of the Xiangxi area. There was no obvious referral preference for gender, while it occurred referral preference for <28 weeks infants. Frequency of CD should be carefully monitored because its use appears to be increasing proportionally each year. The spectrum of neonatal diseases in the NICU in the Xiangxi area was presented, with jaundice, respiratory and neurological diseases being noted as the most prevalent in our sample. The type and prevalence of neonatal disease are closely related to gender, gestational age, patient sources, delivery methods and birth weight. The prevalence of neonatal diseases was significantly higher in males, infants born *via* CD and induced labor, and in infants of lower gestational age and birth weight. The diagnosis and treatment of jaundice, respiratory, and neurological diseases should be improved and more preventive measures formulated for newborns based on gender, gestational age, and birth weight. At the same time, perinatal health care should be further developed in this region—and more generally in China and beyond—to improve the prevention and management of prematurity and infant diseases.

## Data Availability Statement

The original contributions presented in the study are included in the article/supplementary materials, further inquiries can be directed to the corresponding author/s.

## Ethics Statement

The studies involving human participants were reviewed and approved by Biomedical Ethics Committee of Jishou University (JSDX-2021-0028). Written informed consent to participate in this study was provided by the participants' legal guardian/next of kin.

## Author Contributions

FX was involved in the design, data analysis, and writing of the manuscript. YZ, LC, and RH participated in the data acquisition. QS, Z-yC, and JL was involved in the conceptual framing of the manuscript, review of background literature, and editing of the manuscript. All authors read and approved the final draft.

## Funding

This study was supported by Hunan Provincial Innovation Foundation for Postgraduate (ID: CX20211063), the National Social Science Fund of China (ID: 21XMZ090), and Jishou University School-Level Scientific Research Project (ID: Jdlc2015).

## Conflict of Interest

The authors declare that the research was conducted in the absence of any commercial or financial relationships that could be construed as a potential conflict of interest.

## Publisher's Note

All claims expressed in this article are solely those of the authors and do not necessarily represent those of their affiliated organizations, or those of the publisher, the editors and the reviewers. Any product that may be evaluated in this article, or claim that may be made by its manufacturer, is not guaranteed or endorsed by the publisher.
